# Attitudes toward and Uptake of H1N1 Vaccine among Health Care Workers during the 2009 H1N1 Pandemic

**DOI:** 10.1371/journal.pone.0029478

**Published:** 2011-12-22

**Authors:** Joan M. Henriksen Hellyer, Aaron S. DeVries, Sarah M. Jenkins, Kandace A. Lackore, Katherine M. James, Jeanette Y. Ziegenfuss, Gregory A. Poland, Jon C. Tilburt

**Affiliations:** 1 Program in Professionalism and Ethics, Mayo Clinic, Rochester, Minnesota, United States of America; 2 Infectious Disease, Epidemiology, Prevention and Control Division, Minnesota Department of Health, St. Paul, Minnesota, United States of America; 3 Division of Biomedical Statistics and Informatics, Mayo Clinic, Rochester, Minnesota, United States of America; 4 Biomedical Ethics Research Unit, Mayo Clinic, Rochester, Minnesota, United States of America; 5 Division of Health Care Policy and Research, Mayo Clinic, Rochester, Minnesota, United States of America; 6 Survey Research Center, Department of Health Sciences Research, Mayo Clinic, Rochester, Minnesota, United States of America; 7 Mayo Vaccine Research Group, Mayo Clinic, Rochester, Minnesota, United States of America; 8 Program in Translational Immunovirology and Biodefense, Mayo Clinic, Rochester, Minnesota, United States of America; 9 Division of General Internal Medicine, Mayo Clinic, Rochester, Minnesota, United States of America; Centers for Disease Control and Prevention, United States of America

## Abstract

**Background:**

Though recommended by many and mandated by some, influenza vaccination rates among health care workers, even in pandemics, remain below optimal levels. The objective of this study was to assess vaccination uptake, attitudes, and distinguishing characteristics (including doctor-nurse differences) of health care workers who did and did not receive the pandemic H1N1 influenza vaccine in late 2009.

**Methodology/Principal Findings:**

In early 2010 we mailed a self-administered survey to 800 physicians and 800 nurses currently licensed and practicing in Minnesota. 1,073 individuals responded (cooperation rate: 69%). 85% and 62% of Minnesota physicians and nurses, respectively, reported being vaccinated. Accurately estimating the risk of vaccine side effects (OR 2.0; 95% CI 1.5–2.7), agreeing with a professional obligation to be vaccinated (OR 10.1; 95% CI 7.1–14.2), an ethical obligation to follow public health authorities' recommendations (OR 9.9; 95% CI 6.6–14.9), and laws mandating pandemic vaccination (OR 3.1; 95% CI 2.3–4.1) were all independently associated with receiving the H1N1 influenza vaccine.

**Conclusions/Significance:**

While a majority of health care workers in one midwestern state reported receiving the pandemic H1N1 vaccine, physicians and nurses differed significantly in vaccination uptake. Several key attitudes and perceptions may influence health care workers' decisions regarding vaccination. These data inform how states might optimally enlist health care workers' support in achieving vaccination goals during a pandemic.

## Introduction

In April 2009, H1N1 pandemic influenza rapidly spread throughout the world challenging the response capacity of health care organizations and public health authorities. When the influenza A/H1N1 influenza monovalent vaccine became available in October 2009, public health authorities ranked health care personnel as one of the highest priority groups to receive the vaccine [1]. This recommendation was based on health care providers' risk for acquiring infection while caring for patients with influenza, their risk for infecting vulnerable patients if ill with influenza themselves, as well as the public safety need to ensure the presence of adequate personnel to treat an influx of persons ill with influenza. In the absence of a vaccine, initial control strategies for health care workers focused on infection control measures within health care facilities, use of personal protective equipment, establishing triage and other engineered control measures, non-punitive leave policies, and cough and respiratory etiquette to mitigate the pandemic's impact based on past pandemic planning [2], [3].

In Minnesota, preparation for the arrival of the 2009 H1N1 monovalent vaccine began in August of that year [4]. Health care facilities pre-registered with the Minnesota Department of Health (MDH) designated the number of highest priority groups within their facilities including both patients and health care providers. Significant educational efforts regarding the virus and the vaccine were carried out at the state and local levels by public health agencies and health care organizations through electronic messaging, press coverage, online resources, meetings, and webinars, among many other communication modes. 2009 H1N1 influenza activity in Minnesota peaked the week of October 11–17, 2009, with the number of hospitalizations exceeding the previous influenza season of 2008–2009 by over 500% [4]. Around that same time, the vaccine arrived in Minnesota and was distributed to pre-registered health care facilities. By December 2009, there were adequate supplies to meet all requests from health care facilities and most conducted mass vaccination clinics for staff statewide in November and December. As of April 2010, there were 1,824 hospitalized patients and 63 deaths due to laboratory confirmed 2009 H1N1 cases in Minnesota representing only a small fraction of the burden of disease associated with H1N1 influenza [5].

Despite adequate vaccine supply and high priority designation, U.S. health care worker H1N1 vaccination rates were only 37% as of January 2010 [6]. Leading up to the pandemic, the U.S. national annual seasonal influenza vaccination rate among health care workers was estimated to be 45% (ranging from 36% to 52% depending upon health care worker group) over the years 2004–2008 [7].

Previous studies have found that a number of factors influence whether or not health care workers choose to be vaccinated for both seasonal and pandemic influenza, including previous knowledge about the vaccination, risk perception, attitudes toward vaccination such as anticipated regret, perceptions of professional norms, age, and race [8]–[12]. While there have been numerous studies examining the knowledge, attitudes, and practices of professional groups specifically in the context of pandemics [13]–[17], few have examined differences in these areas in a population of U.S. health care workers and across disciplines. Professional groups with distinct cultures and histories may view their perceived obligations toward vaccination during a pandemic differently in light of their particular professional culture or ethos. For instance, the American Medical Association recently adopted a policy that acknowledges physicians' ethical and professional responsibility to be immunized and supporting the requirement that influenza immunization be a condition of initial and continuing employment [18]. The American Nurses Association endorses voluntary immunization and has rejected requirements for influenza immunization of nurses [19].

Following initiatives encouraging health care workers to be vaccinated against 2009 H1N1 influenza, we conducted a state-wide survey of Minnesota physicians and nurses to assess their vaccination behaviors as well as the attitudes and perceptions that may underlie those behaviors, including perceptions of their professional obligations around vaccination and their acceptance of potential mandatory pandemic vaccination policies.

## Materials and Methods

### Ethics Statement

This study was approved by the Mayo Clinic and Minnesota Department of Health Institutional Review Boards (IRBs). The need for written informed consent was waived by both IRBs as data were analyzed anonymously.

### Survey Design and Testing

We devised measures of health care worker vaccination behaviors, knowledge, and attitudes in collaboration with an international team of researchers working on pandemic influenza response. Survey questions were designed with input from content experts in the fields of public health, primary care, and survey methods. Key measures related to professional responsibility were further piloted and cognitively tested with eight clinicians prior to the survey. This involved inviting clinicians who fit our eligibility criteria to complete the survey and provide feedback about challenges they encountered, the time required, and any other positive or negative reactions elicited whilst completing the instrument.

The final questionnaire (see Appendix S1) included broad domains of professional practice, the illness experience, infection control practices, and demographics, as well as items pertaining to vaccination behavior, reasons for accepting or declining vaccination, sources of information about the 2009 H1N1 vaccine, estimation of the frequency of severe side effects associated with H1N1 vaccine, perceptions of ethical obligations to receive vaccination, and perceptions of mandatory vaccination policies.

### Sample & Procedures

Lists of licensed physicians and nurses in Minnesota as of December 2009 were purchased from the State of Minnesota Board of Medical Practice and the Minnesota Board of Nursing. From these lists, we identified all registered nurses in the state and all physicians in specialties providing basic preventive, primary and acute inpatient services (e.g. emergency medicine, geriatric internal medicine, a total of 20 specialties in all; see full list in Appendix S2). Individuals with non-Minnesota addresses were excluded from the frame. From these populations, we randomly selected 800 physicians and 800 nurses. We determined that a 10% difference in immunization rates between nurses and physicians was clinically important. Therefore, assuming a 50% average response rate for healthcare worker surveys, we chose 800 physicians and 800 nurses so that a 10% between-group difference in vaccination rates assuming a 50% response rate (400 returned in each group), with an alpha of .05 (two tailed), would yield approximately 90% power to detect that difference.

In February 2010, we mailed a confidential, self-administered, 7-page questionnaire to these 1,600 health care workers. The initial mailing also included a cover letter, an endorsement letter from the MDH and Mayo Clinic, a postage-paid return envelope, and a laser pointer pen (retail value of $20). Physicians and nurses who did not respond to the first mailing were sent a subsequent mailing six weeks later containing a cover letter, the survey, and a postage-paid return envelope. A third mailing containing these same materials was sent to non-responding physicians and nurses six weeks following the second wave. The administration of each mailing was managed by the Mayo Clinic Survey Research Center.

### Measures of Interest

Our primary outcome variable of interest was respondents' self-reported pandemic H1N1 monovalent influenza vaccination status during the 2009 H1N1 pandemic. Key predictor variables included reasons for accepting or declining vaccination (including personal influenza illness), sources of information about the 2009 H1N1 vaccine, estimation of the frequency of severe side effects associated with H1N1 vaccine, perceived professional obligation to receive the vaccine, perceived ethical obligation to follow public health authorities' recommendations, and endorsement of mandatory vaccination policies. Response categories for these items were either dichotomous or ordinal. For survey items pertaining to reasons for or against vaccination and sources of information about the vaccine, respondents were provided the opportunity to write in reasons and information sources other than those provided as response categories.

### Analyses

Survey results were summarized using frequencies and percentages for categorical responses as well as means and standard deviations for continuous responses. Survey responses were compared between physicians and nurses using Pearson chi-square tests (or Fisher exact tests where appropriate) for categorical responses. Age was compared with the Wilcoxon rank-sum test. Multivariate logistic regression was used to assess the odds of having received the H1N1 influenza vaccination based on respondents' professional occupation (i.e. physician or nurse), perceived professional and ethical obligations, perceived side effects of vaccination, and views about universal vaccination mandates. All models were adjusted for age and sex. P-values less than 0.05 were considered statistically significant. All analyses were performed using SAS version 9.1 (Cary, NC).

## Results

Of the 1,600 sampled Minnesota health professionals, 49 could not be contacted due to undeliverable addresses. Of the remaining 1,551 participants, we received completed surveys from 1,073 for a cooperation rate of 69% [20]. For physicians, 486 of 772 (63%) with deliverable addresses returned completed surveys, while 587 of 779 (75%) nurses with deliverable addresses returned completed surveys.

Respondent characteristics are shown in Table 1. A majority of respondents were female (67%) and the mean age was nearly 48 years. Compared to physicians, nurses were significantly more likely to be female (p<0.001). The majority of both physicians (85%) and nurses (62%) reported receiving vaccination against pandemic H1N1, but physicians were significantly more likely to be vaccinated than nurses (unadjusted p<0.001). Respondents and non-respondents differed in that females were more likely to respond than males (p = 0.001), nurses were significantly more likely to respond than physicians (p<0.001), and those in the metropolitan area of Minneapolis and St. Paul were less likely to respond than those residing in other Minnesota metropolitan or non-metropolitan regions (p<0.001).

**Table 1:** Characteristics and self-reported vaccination behavior of 1073 Minnesota physicians and nurses who completed a survey.

	No. (%)			
Characteristic	Overall	Physicians	Nurses	P-value
	(N = 1073)	(N = 486)	(N = 587)	
Sex				<0.001
Male	355^a^ (33)	316 (66)	39 (7)	
Female	711 (67)	166 (34)	545 (93)	
Age, Mean (SD), y	47.8 (12.5)	48.1 (12.7)	47.5 (12.4)	0.43
Received pandemic H1N1 vaccination	778 (73)	415 (85)	363 (62)	<0.001

a Numbers may not add to the total N due to some missing demographic data.

### Reasons for and against vaccination

Table 2 shows the distribution of responses for why respondents did or did not receive the pandemic H1N1 vaccination. Among those who were vaccinated, the most common reason for vaccination was worry about contracting H1N1 influenza. Physicians who received the H1N1 influenza vaccine were significantly more likely than nurses to select “worry about transmission of swine flu/H1N1 to others” as their primary reason for being vaccinated (28% of physicians vs 20% of nurses, p = 0.01), while vaccinated nurses were slightly more likely than vaccinated physicians to cite “follow the advice from health authorities” (18% of physicians vs 23% of nurses, p = 0.06) as a reason for vaccination.

**Table 2:** Reasons for and against self-reported 2009 H1N1 vaccination behaviors, responses to perceived risk of side effects, and primary sources of information among 486 physicians and 587 nurses from Minnesota.

	No. (%)			
Survey Question & Corresponding Response Options	Overall	Physician	Nurse	P-value for Inter-Professional Difference
If you *have* been vaccinated, which one reason best represents why you were vaccinated?	**(N = 774)**	**(N = 413)**	**(N = 361)**	
Worry about catching swine flu^a^/H1N1 infection	212 (27)	121 (29)	91 (25)	0.23
Worry about transmission of swine flu H1N1 to others	186 (24)	115 (28)	71 (20)	0.01
Follow the advice from health authorities	159 (21)	74 (18)	85 (23)	0.06
Desire to fulfill my professional obligation	111 (14)	53 (13)	58 (16)	0.22
Vaccination is a mandatory requirement in my workplace	51 (7)	31 (8)	20 (6)	0.31
Desire to obtain vaccination early in case of shortage	4 (0.5)	3 (1)	1 (0.3)	0.63
Desire to fulfill the public's expectation	0 (0)	0 (0)	0 (0)	NA
Other reason	34 (4)	14 (3)	20 (6)	0.12
If you were *not* vaccinated, which one reason below represents why you decided not to be vaccinated?	**(N = 290)**	**(N = 70)**	**(N = 220)**	
I don't want to be vaccinated	56 (19)	11 (16)	46 (21)	0.39
Worry about H1N1 vaccine side effects	39 (13)	3 (4)	37 (17)	0.01
Unconcerned about the threat of H1N1 at the moment	35 (12)	10 (14)	25 (11)	0.53
I have contraindications to influenza vaccination	13 (4)	3 (4)	10 (5)	0.99
Universal infection control practices are sufficient	11 (4)	2 (3)	9 (4)	0.99
Now is not the right time; I will be vaccinated at a later stage	10 (3)	5 (7)	5 (2)	0.06
No onsite vaccination service at my workplace	9 (3)	2 (3)	7 (3)	0.99
Worry that H1N1 vaccine might give me flu illness	4 (1)	1 (1)	3 (1)	0.99
I don't think the H1N1 vaccine will work	2 (0.7)	1 (1)	1 (0.4)	0.42
Dislike of the brand of H1N1 vaccine offered	1 (0.3)	0 (0)	1 (0.4)	0.99
Antivirals are more effective than vaccines	1 (0.3)	0 (0)	1 (0.4)	0.99
Which of the following represents your best guess about the frequency of severe side effects associated with the H1N1 vac-cine?	**(N = 1060)**	**(N = 478)**	**(N = 582)**	<0.001
About 1/1,000,000	434 (41)	281 (59)	153 (26)	
About 1/100,000	318 (30)	133 (28)	185 (32)	
About 1/1,000	49 (5)	7 (2)	42 (7)	
I do not know	259 (24)	57 (12)	202 (35)	
What was your primary source of information about the 2009 pandemic H1N1 vaccine?	**(N = 1034)**	**(N = 472)**	**(N = 562)**	
Information from my employer	478 (46)	183 (39)	295 (53)	<0.001
Information from the public health authority	249 (24)	146 (31)	103 (18)	<0.001
Mass media	135 (13)	35 (7)	100 (20)	<0.001
Scientific publications	39 (4)	34 (7)	5 (1)	<0.001
Web sites of health agencies	116 (11)	69 (15)	47 (8)	<0.001
Did not receive any information	3 (0.3)	2 (0.4)	1 (0.2)	0.59
Other	14 (1)	3 (0.6)	11 (2)	0.10

a This term was included in the questionnaire because of its common use in the media at the time.

Parenthetical sample size numbers provided for each item reflect the actual number of respondents who answered that item in the survey.

Among those who were *not* vaccinated, both physicians and nurses most often cited personal choice (i.e. “I don't want to be vaccinated”) as their primary reason for not receiving a vaccination. Nurses were significantly more likely than physicians to have selected “worry about H1N1 vaccine side effects” as their primary reason for not being vaccinated (17% of nurses vs 4% of physicians, p = 0.01).

### Sources of Information and Perceived Side Effects

The most frequently cited source of information about the 2009 H1N1 influenza vaccine by physicians and nurses overall was information from employers (46%), with nurses endorsing this option more frequently than physicians (53% vs 39%, p<0.001). Physicians and nurses differed for nearly all response options in the sources of information category (Table 2). Physicians were more likely than nurses to cite publications by public health authorities (31% versus 18%, p<0.001) and health agency web sites (15% versus 8%, p<0.001) as their primary sources of information about H1N1 vaccination. Nurses, however, were more likely than physicians to cite mass media as their primary source of information about the vaccine (20% versus 7%, p<0.001).

When asked to give their “best guess” regarding the frequency of severe side effects associated with the H1N1 vaccine (the true rate of which can be conservatively estimated at around 1/1,000,000), many fewer nurses than physicians accurately estimated the risk of serious side effects from the vaccination (26% vs. 59%, p = 0.01) (Table 2).

### Professional Obligations

**Figure 1:**
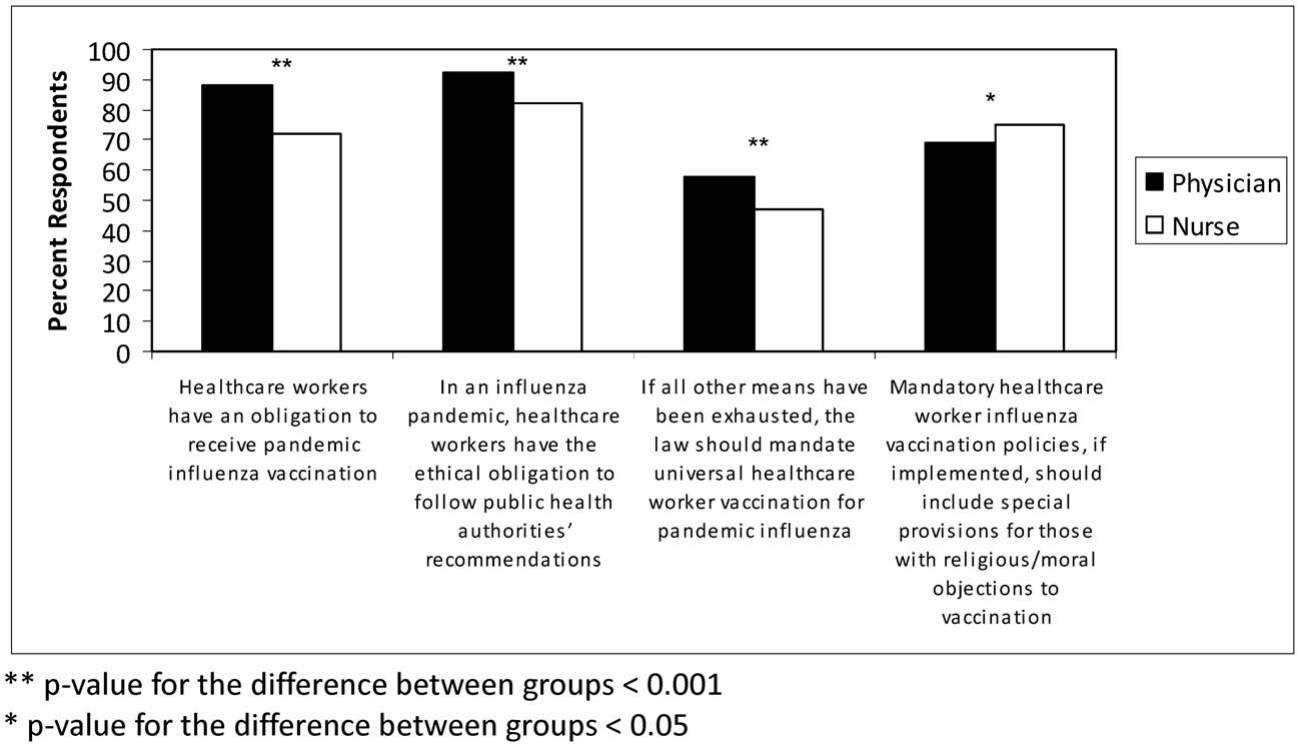
Minnesota health care workers' views of obligations and mandates. Comparison of the distribution of 486 physicians and 587 nurses in a Minnesota survey who agreed with statements regarding obligations to be vaccinated, to follow public health authorities' recommendations, and the permissibility of a health care worker vaccine mandate.

A majority of both professional groups agreed that there is a professional obligation to be vaccinated especially in a pandemic (Figure 1). Compared to nurses, more physicians agreed that “health care workers have a professional obligation to be vaccinated” (88% vs. 72%, p<0.001) and that “in an influenza pandemic, health care workers have an ethical obligation to follow public health authorities' recommendations” (92% vs. 82%, p<0.001). Physicians and nurses were more divided on the topic of vaccination mandates, however. Compared to nurses, more physicians agreed that “if all other means have been exhausted, the law should mandate universal health care worker vaccination for pandemic influenza” (58% vs. 47%, p<0.001).

### Multivariate Model

A multivariable logistic regression model was used to estimate the associations between odds of vaccination with profession, perceived risk of side effects, agreement/disagreement with professional or ethical obligations, and agreement/disagreement with a legal mandate for health care workers to be vaccinated (adjusted for age and sex). After controlling for age and sex, being a physician (OR 3.4; 95% CI 2.3–5.0), accurately estimating the risk of vaccine side effects (OR 2.0; 95% CI 1.5–2.7), agreeing with a professional obligation to be vaccinated (OR 10.1; 95% CI 7.1–14.2), agreeing with an ethical obligation to follow public health authorities' recommendations (OR 9.9; 95% CI 6.6–14.9), and agreeing with laws mandating vaccination (OR 3.1; 95% CI 2.3–4.1) were all independently associated with receiving the H1N1 influenza vaccine (Table 3).

**Table 3:** Multivariate association between profession, perceived professional obligation to receive 2009 H1N1 influenza vaccination, perceived ethical obligation to follow public health authorities' recommendations, perceived risk of side effects, and self-reported 2009 H1N1 influenza vaccination among 1073 Minnesota health care workers.

	Odds of receiving vaccination	
Predictor	OR^a^ (95% CI)	P-value
Professional group		
Nurses	1.0	
Physicians	3.4 (2.3–5.0)	<0.0001
Perceived risk of side effects		
High risk (About 1/100,000 or 1/1,000) or Don't know	1.0	
Low risk (1/1,000,000)	2.0 (1.4–2.5)	<0.0001
Health care workers have a professional obligation to receive vaccination		
Disagree	1.0	
Agree	10.1 (7.1–14.2)	<0.0001
In an influenza pandemic, health care workers have an ethical obligation to follow public health authorities' recommendations		
Disagree	1.0	
Agree	9.9 (6.6–14.9)	<0.0001
The law should mandate universal health care worker vaccination for pandemic influenza		
Disagree	1.0	
Agree	3.1 (2.3–4.1)	<0.0001

a Adjusted for all other predictors in the model as well as age and sex.

## Discussion

These data from a state-wide survey of health professionals in the wake of the 2009 H1N1 pandemic suggest high (but not universal) rates of vaccination in key health care worker populations and several key attitudes and perceptions that may have influenced whether health care professionals were vaccinated. Chief among these associations include perceptions about risk of serious side effects, professional obligations, and sources of information, all of which varied by professional group. These data raise important questions about inter-professional education and differences in response to pandemics and how states might enlist the optimal support of health care workers in achieving vaccination targets during pandemics.

The self-reported vaccination rates of 85% for physicians and 62% for nurses reported here are among the highest we have seen in population-based assessments of voluntary vaccination programs, such as the 13.5%, 36.2%, 36.5%, and 41.3% rates seen in Hong Kong, Singapore, France, and the United Kingdom, respectively [17], [21]. Compared to early reports of health care worker seasonal vaccination rates as of November 2010, self-reported rates of pandemic vaccination from 2009 may represent a best case scenario of vaccination rates in which pandemic concerns combined with shortages and intense public appetite for the vaccine created a temporary enthusiasm for vaccination in response to an extraordinary threat [6].

Recent surveys of Canadian, English, French, and Spanish health care workers also found concern over vaccine side effects to be one of the top reasons workers declined to receive the H1N1 influenza vaccine [10], [16], [17], [22]. The actual risk of serious side effects associated with the influenza vaccine is estimated to be one event per 4–8 million doses based on data from the Vaccine Adverse Events Reporting System [23]. We found that physicians were more likely to most closely estimate the order of magnitude of the risk (i.e. 1/1,000,000) for pandemic vaccine side effects compared to nurses who were more likely to respond “I do not know” or to over-estimate the risk. The absolute risk of serious vaccine side effects for influenza vaccine is similar to the risk associated with hepatitis B vaccine, a vaccine that health care institutions are required to track and offer free of charge for all employees [24]. This illustrates the importance of accurate education to health care providers regarding both level of risk and vaccine efficacy.

Our finding that lower rates of vaccination among nurses was associated with an inaccurate perceived estimate of vaccine side effects also mirrors those by Zhang et al in their survey of nurses in the United Kingdom [12]. In that study, nurses' over-estimation of the seasonal influenza vaccine's side effects and less frequent use of public health authorities for vaccine information suggests that public health messaging either does not sufficiently target nurses or uses communication channels and strategies that may not be optimally effective. For instance, suboptimal trust by nurses of their employers because of labor concerns may translate to suboptimal trust in public health messages about vaccination communicated through employers [25]. Such may be the case for Minnesota nurses, in whom major labor negotiations and strike threats were occurring in the winter of 2009–2010 concurrent with the pandemic [26]. Although this study by Zhang et al. focused on nurses' perceptions of seasonal, as opposed to pandemic, influenza vaccination side effects, the findings nevertheless provide important context for the relevant and related scenario of pandemic vaccination.

That nurses are less likely to view vaccination as a professional obligation raises the possibility that either fewer nurses do not consider such actions as central to their professional identity, or that the way they view their professional role is not fundamentally about “obligations” to perform certain actions *per se*, but rather about fidelity to particular individual caring relationships. Although not directly addressed by this survey, nurses may endorse strong professional norms related to patient safety primarily in the context of particular relationships.

From these data we speculate on several possible implications for public health and organizational practice. Pandemic influenza messaging may need to target nurses more directly with facts about the significant benefits of vaccination for themselves and their patients in comparison to the associated extremely low risk of negative side effects. These data also raise questions about professional differences in the sources of professional identity generally (i.e. what it means to be a nurse or physician). While aspirational ethical statements may establish a professional obligation to receive vaccinations, as these data suggest, direct appeals to nurses' sense of general professional obligation may not be the most effective framework for bringing about behavior change. Rather, we speculate that focusing on caring relationships as a source of professional behavior change may be more in line with nurses' professional self-identity. For example, perhaps messaging campaigns could appeal directly to nurses' sense of advocacy for the many vulnerable patients they care for daily, as a means of promoting vaccination in a manner more in line with their professional identity. Such efforts deserve further investigation.

Whether health care worker mandates should be legislated is an ongoing policy question that these data address indirectly. While some respondents voiced objections to mandates, recent data suggests that mandates are generally well accepted once in place [27], [28]. Mandates may be more likely to be accepted by doctors and nurses who claimed they did not receive the vaccination because they “don't want to be vaccinated,” simply because they otherwise voice no strong reasons for avoiding it [29]. More nurses may view vaccination as a personal health choice rather than an obligatory patient safety measure [30]. If so, it is easier to see how they may not find the time to fit in a “personal health choice” as a priority in a busy life. Despite disagreement about endorsement for mandates, the percent of health care workers who cite reasons for not being vaccinated due to personal choice was low (16% physicians and 21% nurses).

These data have several limitations. First, the sample was limited not only to the United States context, but specifically one state – a state in which there is generally strong public health infrastructure and coordinated responses to public health emergencies. Our respondent population was also comprised of more women than men, and more nurses than physicians – characteristics which may limit the representativeness of the findings for the health care worker population as a whole. The use of survey data also raises the possibility of response bias if responders systematically differ from non-responders on the characteristics being studied. Further, self-report can be subject to measurement error due to how items were worded. We cannot exclude the possibility of a social desirability bias for such a sensitive topic whereby respondents answer in a way that will cast them in a more positive light. Using a self-administered survey without the presence of an interviewer somewhat minimizes this concern. Moreover, our relatively high response rate and explicitly stated commitment to confidentiality should have minimized this.

Furthermore, our approach (i.e. surveying with closed-ended items) limited our ability to explore the actual concepts such as professional obligation that nurses and physicians may use to form judgments on these matters. Such an approach like that used by Rhudy et al may be warranted in future studies [30]. Finally, cross-sectional measurements cannot establish causation between predictors and outcomes. Despite these limitations, however, these data suggest that a variety of perceived risks, professional norms, information sources, and professional identity may impact how health care workers respond optimally to pandemic action plans at the state level. Crafting state-level responses to pandemics that account for potential misperceptions about vaccine risks and side effects, as well as inter-professional differences, may enhance the ability of the public health workforce to meet the challenges of the next influenza pandemic.

## Supporting Information

Appendix S1 **Copy of survey administered to Minnesota health care workers.**
(PDF)

Appendix S2 **Specialties providing basic preventive, primary and acute inpatient services among which 800 Minnesota physicians were chosen to receive a survey.**
(DOC)
