# Supplementary material for: Attitudes toward and Uptake of H1N1 Vaccine among Health Care Workers during the 2009 H1N1 Pandemic
Source: PLoS One. 2011 Dec 22;6(12):e29478. doi: 10.1371/journal.pone.0029478 (PMC3245279; doi:10.1371/journal.pone.0029478)
Supplement: Appendix S1 — Copy of survey administered to Minnesota health care workers. (PDF) [file pone.0029478.s001.pdf]

Appendix S1

Healthcare Professionals' Perspectives on Pandemic H1N1 (Swine Flu)

___ ___ ___ ___

INSTRUCTIONS: PLEASE CHECK THE APPROPRIATE BOX OR FILL IN THE BLANK AS INDICATED.

Your Professional Practice

1. What is your PRIMARY site of work? (Mark <u>ONE</u> only.)

1 ☐ Outpatient private practice clinic
2 ☐ Outpatient community health center/public clinic
3 ☐ Hospital emergency room
4 ☐ Urgent care center (for US)
5 ☐ Other, please specify: ____________________

2. Which of the following best describes your position? (Mark <u>ONE</u> only.)

1 ☐ Internal medicine
2 ☐ Family medicine
3 ☐ Emergency medicine (casualty)
4 ☐ Pediatrics
5 ☐ General outpatient clinic
6 ☐ Surgery
7 ☐ Physiotherapy/occupational therapy/dietitian
8 ☐ Non-clinical
9 ☐ Administration
10 ☐ Critical care
11 ☐ Other, please specify: ____________________

3. What is your major job title/duty? (Mark <u>ONE</u> only.)

1 ☐ Physician (MD, DO)
2 ☐ Nurse (EN, RN, LPN)
3 ☐ Nurse-Practitioner
4 ☐ Physician assistant
5 ☐ Allied health, please specify: ____________________
6 ☐ Administration
7 ☐ Other, please specify: ____________________

4. How many years have you been working in healthcare after graduation?

1 ☐ Less than or equal to 5 years
2 ☐ 6 to 10 years
3 ☐ 11 to 20 years
4 ☐ More than 20 years

5. With how many patients do you have direct contact (e.g., face-to-face conversation) in a typical week?

1 ☐ 0
2 ☐ 1 to 25
3 ☐ 26 to 50
4 ☐ More than 50

Your Illness Experience

6. Since April 30, 2009, have you had an influenza-like illness (fever greater than 100°F [37.8°C] and cough or sore throat)?

1 ☐ No   2 ☐ Yes

Was this illness determined to be:

1 ☐ Seasonal influenza
2 ☐ 2009 pandemic H1N1 (swine flu)
3 ☐ Other, please specify: ______________________
4 ☐ No confirmed diagnosis

How was the influenza diagnosed?

1 ☐ By symptoms alone   4 ☐ PCR
2 ☐ Rapid influenza test   5 ☐ Other, please specify: ________
3 ☐ Viral culture   6 ☐ Not sure

7. How likely do you think you are to contract 2009 pandemic H1N1 influenza (swine flu) during the next wave (assuming you have not been vaccinated)? (Please answer even if you have been infected during the previous wave.)

1 ☐ Very unlikely   2 ☐ Unlikely   3 ☐ Likely   4 ☐ Very likely

8. If you were to contract 2009 pandemic H1N1 influenza (swine flu) today, how seriously do you think your illness would affect your health? (Please answer even if you have already contracted 2009 pandemic H1N1 influenza [swine flu].)

1 ☐ Not at all serious   2 ☐ Slightly serious   3 ☐ Moderately serious   4 ☐ Very serious

9. Which of the following represents your best guess about the frequency of severe side effects (e.g., Guillain-Barre syndrome) associated with the H1N1 vaccine?
(Mark **ONE** only.)

1 ☐ About 1/1,000,000 (not more than a few cases per million vaccinated)
2 ☐ About 1/100,000 (not more than a few cases per one hundred thousand vaccinated)
3 ☐ About 1/1,000 (not more than a few cases per thousand vaccinated)
4 ☐ I do not know

Infection Control Practices

10. Do you currently have regular direct contact (e.g., face-to-face conversation) with patients in your day-to-day work?

1 ☐ No — **Skip to question 11 below.**

2 ☐ Yes

**Since April 2009, <u>when having contact with patients you suspect of having symptoms of influenza (seasonal or pandemic)</u>, how often do you employ the following practices?**	Never	Seldom	Sometimes	Always
**Wear gloves during physical examination/physical contact**	1 ☐	2 ☐	3 ☐	4 ☐
**Change gloves after each patient contact**	1 ☐	2 ☐	3 ☐	4 ☐
**Wash hands <u>before</u> each patient contact**	1 ☐	2 ☐	3 ☐	4 ☐
**Wash hands <u>after</u> each patient contact**	1 ☐	2 ☐	3 ☐	4 ☐
**Wear an N95 respiratory (TB mask)/surgical mask (as recommended by your institute) during physical examination/physical contact**	1 ☐	2 ☐	3 ☐	4 ☐
**Wear a surgical mask in clinic or ward during a pandemic even when not involved in direct patient care**	1 ☐	2 ☐	3 ☐	4 ☐

Influenza Vaccinations

11. Have you received the <u>seasonal</u> influenza vaccine for the current season (late 2009)?

1 ☐ No 2 ☐ Yes

12. Have you received the <u>seasonal</u> influenza vaccine within the last 5 years?

1 ☐ No 2 ☐ Yes

If yes, how many times? ____ (Number)

13. Have you received the <u>2009 pandemic H1N1</u> (swine flu) vaccine?

1 ☐ No

If no, which one reason below BEST represents why you decided NOT to be vaccinated? (Mark **<u>ONE</u>** only.)

1 ☐ Now is NOT the right time; I will be vaccinated at a later stage
2 ☐ Unconcerned about the threat of H1N1 at the moment
3 ☐ I don't think the H1N1 vaccine will work
4 ☐ Worry about H1N1 vaccine side effects
5 ☐ Worry that H1N1 vaccine might give me flu illness
5 ☐ Dislike of the brand of H1N1 vaccine offered
6 ☐ Antivirals are more effective than vaccines
7 ☐ Universal infection control practices are sufficient
8 ☐ No onsite vaccination service at my workplace
9 ☐ I don't want to be vaccinated
10 ☐ I have contraindications to influenza vaccination
11 ☐ Other, please specify: ______________________

2 ☐ Yes

If yes, which one reason below BEST represents why you were vaccinated? (Mark **<u>ONE</u>** only.)

1 ☐ Worry about catching swine flu H1N1 infection
2 ☐ Worry about transmission of swine flu H1N1 to others
3 ☐ Desire to obtain vaccine early in case of shortage
4 ☐ Follow the advice from health authorities
5 ☐ Vaccination is a mandatory requirement in my workplace
6 ☐ Desire to fulfill the public's expectation
7 ☐ Desire to fulfill my professional obligation
8 ☐ Other, please specify: ______________________

14. Did you obtain information about the 2009 pandemic H1N1 (swine flu) vaccine from any of the following sources? (Mark "yes" or "no" for each, and of those you mark yes, please indicate which ONE was your <u>primary</u> source.)

	No	Yes	**Which one was your primary source?** (Mark one.)
**Information from my employer**	1 ☐	2 ☐	1 ☐
**Information from the public health authority**	1 ☐	2 ☐	2 ☐
**Mass media**	1 ☐	2 ☐	3 ☐
**Scientific publications**	1 ☐	2 ☐	4 ☐
**Web sites of health agencies**	1 ☐	2 ☐	5 ☐
**Did not receive any information**	1 ☐	2 ☐	6 ☐
**Other, please specify:** ______________	1 ☐	2 ☐	7 ☐

15. In your opinion, please rank in order of priority the healthcare worker groups you think should be vaccinated first in a pandemic. (1 = highest priority, 2 = 2nd highest, 3 = 3rd highest, and so on.)

_____ Administration

_____ Allied health (physiotherapists, radiographers, occupational therapists, dietitians)

_____ Doctors/nurse practitioners/physician assistants

_____ Non-clinical

_____ Nurses

16. In your opinion, should healthcare workers who have been offered and declined H1N1 vaccination (and are not part of an allowable exception group) play a different professional role than their usual role during a pandemic?
(Mark **ONE** only.)

1 ☐ No, they should perform their regular duties
2 ☐ Yes, they should be reassigned to non-patient care activities
3 ☐ Other, please specify:

____

____

17. Please indicate your agreement or disagreement with the following statements.

	Strongly disagree	Moderately disagree	Moderately agree	Strongly agree
**If all other means have been exhausted, the law should mandate <u>universal</u> healthcare worker vaccination for <u>seasonal</u> influenza.**	1 ☐	2 ☐	3 ☐	4 ☐
**If all other means have been exhausted, the law should mandate <u>universal</u> healthcare worker vaccination for <u>pandemic</u> influenza.**	1 ☐	2 ☐	3 ☐	4 ☐
**Healthcare workers have an obligation to receive pandemic influenza vaccination.**	1 ☐	2 ☐	3 ☐	4 ☐
**In an influenza pandemic, healthcare workers have the ethical obligation to follow public health authorities' recommendations**	1 ☐	2 ☐	3 ☐	4 ☐
**Mandatory healthcare worker influenza vaccination policies, if implemented, should include special provisions for those with religious/moral objections to vaccination.**	1 ☐	2 ☐	3 ☐	4 ☐

Demographic Information

18. What is your age?

___ ___ Years

19. What is your gender?

1 ☐ Male 2 ☐ Female

20. In what year did you graduate from nursing/medical school/healthcare institute?

___ ___ ___ ___ Year

Thank you very much!

Please return this survey in the envelope provided.
