# Supplementary material for: Attitudes toward and Uptake of H1N1 Vaccine among Health Care Workers during the 2009 H1N1 Pandemic
Source: PLoS One. 2011 Dec 22;6(12):e29478. doi: 10.1371/journal.pone.0029478 (PMC3245279; doi:10.1371/journal.pone.0029478)
Supplement: Appendix S2 — Specialties providing basic preventive, primary and acute inpatient services among which 800 Minnesota physicians were chosen to receive a survey. (DOC) [file pone.0029478.s002.doc]

**Appendix S2.**

Adolescent Medicine

Aerospace Medicine

Anesthesiology

Cardiology

Cardiovascular Disease

Critical Care Medicine

Emergency Medicine

Family Medicine

General Preventive Medicine

Geriatric Medicine

Geriatric Medicine-FP

Geriatrics-Internal Medicine

Infectious Disease

Internal Medicine

Pediatric Emergency Medicine

Pediatrics

Public Health & General Preventive Medicine

Pulmonary Disease

Surgery-General

Surgical Critical Care
